# Electronic cigarette use in Greece: an analysis of a representative population sample in Attica prefecture

**DOI:** 10.1186/s12954-018-0229-7

**Published:** 2018-04-13

**Authors:** Konstantinos E. Farsalinos, Georgios Siakas, Konstantinos Poulas, Vassilis Voudris, Kyriakoula Merakou, Anastasia Barbouni

**Affiliations:** 1 Onassis Cardiac Surgery Center, Sygrou 356, 17674 Kallithea, Greece; 2 Department of Pharmacy, University of Patras, 26500 Rio, Greece; 3 National School of Public Health, Alexandras Av. 196, 11521 Athens, Greece; 4 Public Opinion Research Unit, University of Macedonia, Egnatia 156, 546 36 Thessaloniki, Greece

**Keywords:** Electronic cigarettes, Smoking/harm reduction, Nicotine, Greece

## Abstract

**Background:**

The purpose was to assess prevalence and correlates of electronic cigarette (e-cigarette) use in Greece in 2017.

**Methods:**

A cross-sectional survey of a representative sample of 4058 adults living in Attica prefecture (35% of the Greek adult population) was performed in May 2017 through telephone interviews. Prevalence and frequency of e-cigarette use were assessed according to the smoking status, and logistic regression analysis was performed to identify correlates of use.

**Results:**

Current smoking was reported by 32.6% of participants. Ever e-cigarette use was reported by 54.1% (51.4–56.8%) of current smokers, 24.1% (21.7–26.5%) of former smokers and 6.5% (5.3–7.7%) of never smokers. Past experimentation was the most prevalent pattern of e-cigarette use among ever users (*P* < 0.001). Almost 80% of ever and 90% of current e-cigarette users were using nicotine. Extrapolated to the whole Attica population (3.1 million), there were 1 million current smokers, 848,000 ever e-cigarette users and 155,000 current e-cigarette users. The majority of current e-cigarette users (62.2%) were former smokers. Only 0.2% of never smokers were current e-cigarette users. One out of 20 participants considered e-cigarettes a lot less harmful than smoking. Being current or former smoker were the strongest correlates current e-cigarette use (OR 30.82, 95%CI 10. 21–69.33 and OR 69.33, 95%CI 23.12–207.90 respectively).

**Conclusions:**

E-cigarette use in Greece is largely confined to current or former smokers, while current use and nicotine use by never smokers is extremely rare. The majority of current e-cigarette users were former smokers. Most participants overestimate the harmfulness of e-cigarettes relative to smoking.

**Electronic supplementary material:**

The online version of this article (10.1186/s12954-018-0229-7) contains supplementary material, which is available to authorized users.

## Background

Electronic cigarettes (e-cigarettes) have been increasingly popular over the past few years, and this has been a matter of intense debate within the public health community. Two major areas of concern are their safety/risk profile and patterns of use by the population. For the former, currently available evidence suggests that they are by far less harmful than smoking; however, these estimates are mainly derived from chemistry and toxicology studies, and there is still no long-term epidemiological evidence to accurately quantify the level of risk reduction [1–4]. Moreover, e-cigarettes emit some toxins, although at low levels, suggesting that they are not absolutely safe. The use and popularity of e-cigarettes among various population subgroups can raise important issues. From a public health perspective, e-cigarette use would be desirable if it was confined to smokers and if the intended use would be as substitute for smoking. Additionally, it is important to assess whether e-cigarette use promotes smoking cessation. On the contrary, use by non-smokers would result in exposure to toxins which could confer some additional health risk compared to not using any inhalational product. Therefore, assessing the patterns of e-cigarette use in the population, focusing on the smoking status of users, is particularly important in determining the public health impact of e-cigarettes [3].

Few randomized controlled trials assessing the efficacy of e-cigarettes in smoking cessation and reduction have shown modest results [5–7]. A metanalysis of such studies has shown that e-cigarettes are effective in smoking cessation, but the level of evidence was low [8]. Several cohort studies have shown mixed results [9–12], which was also reflected in metanalyses showing that e-cigarettes promote, have no effect or prevent smoking cessation [13–15]. Several concerns have been raised about these studies, mainly methodological issues that create substantial bias and make many of the studies unsuitable for evaluating the effects of e-cigarettes on smoking cessation [16]. A review of studies evaluating e-cigarettes effects on smoking cessation identified that only a small proportion met the proposed quality criteria [16]. Additionally, randomized controlled trials have inherent problems, such as the long duration for trial planning, recruitment, implementation and analysis [17]. Thus, they may not be the best way of assessing a behavioural intervention such as e-cigarette use, with the large variability of devices and flavours being used according to self-preference by consumers. In this context, real world and population studies provide a useful insight on the impact of e-cigarettes on smoking prevalence. In the UK, the increase in prevalence of e-cigarette use by smokers was positively associated with the success rates of quit attempts and the association remained significant after adjustment for a range of confounding variables [18]. In their latest report, Public Health England (PHE) estimated that e-cigarettes have contributed up to 57,000 additional former smokers in 2016 in the UK [19].

Greece historically has a high prevalence of smoking, ranked among the highest within the European Union [20]. In 2002, the prevalence of smoking was 39.2% in the EU and 42% in Greece [21]. By 2014, the smoking prevalence was reduced to 26% in the EU (a 34% relative reduction) but remained quite high in Greece (38%, a 10% relative reduction) [20]. While e-cigarettes appear to have become popular in Greece, no published studies have provided any data on the true prevalence of e-cigarette use and the patterns of use by different population subgroups. For this reason, a cross-sectional study was performed in 2017, evaluating smoking and e-cigarette use in adult inhabitants of the Attica prefecture, the largest prefecture of Greece. The main aims of this study were to assess the (1) e-cigarette awareness and prevalence of ever, current and past e-cigarette use, as well as past experimentation; (2) prevalence of nicotine-containing e-cigarette use; and (3) correlates of ever and current e-cigarette use.

## Methods

### Design, setting and participants

The cross-sectional study was performed in May 2017 in a sample of 4058 respondents aged ≥18 years. The population of the survey was inhabitants in the Attica prefecture. This region hosts 35% of the total adult population of Greece according to Census 2011 (http://www.statistics.gr/en/2011-census-pop-hous) and consists of the Regional Departments of Athens, Piraeus, Eastern Attica and Western Attica. The sample drawn was representative of the prefecture of Attica population, with all registered landline phone numbers being used as the sampling frame. According to the Hellenic Authority for Telecommunications and Postal services (www.eett.gr) landline phones penetration in 2016 was 44.1% of the population, while mobile phones was 148%. The above difference is a common example for a possible coverage error [22]. For the landline phones, the potential issue on the survey is the under-coverage, but for the usage of mobiles phones, it is the over-coverage. Unfortunately, there is no reliable benchmark study for the Greek population that identifies the direction, strength and potential implications of the coverage error. Using the landline phones prevented the error of over-presenting into the sample people that own more than one mobile phone. The mode of data collection was computer-assisted telephone interviews (CATI), and the sample design was stratified random sampling. Further post-survey adjustment weights were applied in order to depict sample composition as the actual population demographics.

The study was approved by the ethics committee of the Onassis Cardiac Surgery Center (reference no. 591/14.12.16). All participants provided verbal consent at the beginning of the interview in order to participate to this study.

### Sociodemographic factors

The exact age of the participants was recorded and then recoded into five categories (18–24, 25–39, 40–54 and 55 years and older). Education was coded as “high school or less”, “technical education”, “university education” and “postgraduate education”. Marital status was recorded and categorized as “single”, “married/living with a partner” and “widowed/divorced”. The financial status of the participants was assessed through a question seeking a self-assessment report of the financial conditions of each household: “How would you characterize your financial situation?”. Response options were “We are unable to cope with our household finances” (very bad financial situation), “We are able to cope with our household finances, but with a lot of difficulties” (bad financial situation), “We are able to to cope with our household finances, but without the ability to save a lot of money” (not good financial situation), and “We don’t have financial problems” (good financial situation). This variable attempts to accommodate measurement issues, processing errors and item non-response errors [22, 23]. For the analysis, responses were recoded into three groups (“very bad/bad”, “not good”, and “good”).

### Smoking and e-cigarette use

Participants were categorized according to their smoking status as current smokers, past smokers and never smokers. Current and former smokers were asked whether they were smoking daily or occasionally. Current daily smokers were asked whether they had tried to quit smoking in the past 3 years and, if yes, which smoking cessation methods they had used. Response options were “Nicotine replacement therapies”, “Oral smoking cessation medications”, “Psychological support”, “E-cigarettes” and “By myself (without any aid)”, with participants being able to choose multiple responses.

E-cigarette awareness was assessed by asking: “Have you ever heard of e-cigarettes?”. Those responding “No” were classified as never e-cigarette users and no further questions about e-cigarettes were asked. E-cigarette use was assessed by asking: “Regarding the use of e-cigarettes, which of the following statements applies to you?” The responses “I currently use e-cigarettes” (current use), “I used them in the past, but no longer use them” (past use) and “I tried them in the past only once or twice” (past experimentation) were categorized as ever e-cigarette use. The response “I have never used them” was categorized as never use. A separate question assessed the use of nicotine-containing e-cigarettes among ever e-cigarette users, by asking “Do/did you use nicotine in your e-cigarette?” Current and past e-cigarette users were asked whether they use or used the e-cigarette daily or occasionally. The harm perception for e-cigarettes was examined by asking “Do you think e-cigarettes are…”, with available responses being “A lot more harmful than tobacco cigarettes”, “More harmful than tobacco cigarettes”, “As harmful as tobacco cigarettes”, “Less harmful than tobacco cigarettes”, “A lot less harmful than tobacco cigarettes”, “Absolutely harmless” and “Don’t know”.

### Statistical analysis

All values were presented as weighted proportions with 95%CI, while the projected number within the Attica population was extrapolated based on the total sampling population using an appropriate weighing variable. Differences in prevalence of e-cigarette current use, past use and past experimentation were assessed using chi-square (*χ*^2^) test. Two separate logistic regression analyses were performed to assess correlates of ever and current e-cigarette use, similarly to a previous analysis of data from Eurobarometer 2014 [24]. Independent variables included all demographics as well as the perception of e-cigarette harmfulness. The latter was recoded as a binary variable, with responses “A lot more harmful than tobacco cigarettes”, “More harmful than tobacco cigarettes”, “As harmful as tobacco cigarettes” and “Don’t know” coded as 1 and “Less harmful than tobacco cigarettes”, “A lot less harmful than tobacco cigarettes” and “Absolutely harmless” coded as 2. All analyses were weighted and were performed with commercially available software (SPSS v.22.0, Chicago, IL, USA).

## Results

### Smoking and e-cigarette use in the population of Attica

Participant demographics are presented in Additional file 1: Table S1. The prevalence of smoking and e-cigarette use is shown in Table 1. Current smoking was reported by 32.6% of the population while an additional 29.7% were former smokers. The majority were daily smokers (85.8% for current smokers and 76.9% for former smokers). Cessation attempts over the past 3 years were reported by 46.9% (95%CI 44.0–49.8%) of current daily smokers. Among these, quitting without any aid was the most popular method (74.6%, 95%CI 70.9–78.3%), followed by e-cigarettes (47.4%, 95%CI 43.1–51.6%), nicotine replacement therapies (13.0%, 95%CI 10.1–15.8%) and oral smoking cessation medications (4.9%, 95%CI 3.1–6.7%). Extrapolated to the total Attica population, there were 194,000 current daily smokers who had used e-cigarettes in a smoking cessation attempt compared to 53,000 using nicotine replacement therapies and 20,000 using oral smoking cessation medications.

**Table 1:** Smoking and e-cigarette use in the total sample

Characteristic	*N* (unweighted)	% (95%CI) (weighted)
Smoking		
Current smokers	1281	32.6% (31.2–34.0%)
Former smokers	1287	29.7% (28.3–31.1%)
Never smokers	1490	37.7% (36.2–39.2%)
Smoking frequency^1^		
Daily smokers	2136	81.6% (80.1–83.1%)
Occasional smokers	432	18.4% (16.9–19.9%)
E-cigarette awareness		
Yes	3642	91.7% (90.9–92.5%)
No	407	8.1% (7.3–8.9%)
E-cigarette use		
Ever use	924	27.2% (25.8–28.6%)
Current use	169	5.0% (4.3–5.7%)
Past use	374	9.8% (8.9–10.7%)
Past experimentation	381	12.4% (11.4–13.4%)
Never use^2^	3124	72.7% (71.3–74.1%)
Frequency of e-cigarette use^3^		
Daily use	435	79.5% (76.6–82.7%)
Occasional use	106	20.2% (17.0–23.4%)
Nicotine-containing e-cigarette use^4^	720	77.9% (75.5–80.3%)

^1^Proportion of current and former smokers

^2^Includes participants who are not aware of e-cigarettes

^3^Proportion of current and past e-cigarette users

^4^Proportion of ever e-cigarette users

More than 9 out of 10 participants were aware of e-cigarettes. Ever e-cigarette use was reported by 27.2% of the population. Current e-cigarette use was reported by 5.0% of the population, while 1 out of 10 participants reported being past e-cigarette users. The majority were daily e-cigarette users (78.8% for current and 79.9% for past e-cigarette users). Past experimentation was the most prevalent pattern of e-cigarette use for the whole population of ever e-cigarette users (*χ*^2^ = 142.5, *P* < 0.001). Nicotine use was reported by the majority of ever e-cigarette users and was more prevalent in current (88.9%) and past e-cigarette users (84.0%) compared to past experimenters (68.6%; *χ*^2^ = 61.8, *P* < 0.001). Extrapolated to the total population of 3.1 million adult inhabitants in Attica, there were 1 million current smokers, 848,000 ever e-cigarette users and 155,000 current e-cigarette users at the time of the survey.

### E-cigarette use according to the smoking status

The prevalence of e-cigarette use according to the smoking status is shown in Table 2, including data on daily use and nicotine-containing e-cigarette use. From the total population of ever e-cigarette users, 64.8% were current smokers, 26.2% were former smokers and 9.0% were never smokers. From the total population of current e-cigarette users, 36.3% were current smokers, 62.2% were former smokers and 1.5% were never smokers.

**Table 2:** E-cigarette use according to the smoking status

	Smoking status		
	Current smokers	Former smokers	Never smokers
	% (95%CI) (weighted)		
E-cigarette use			
Current use	5.5% (4.3–6.7%)	10.4% (8.7–12.1%)	0.2% (0.0–0.4%)
Daily use	3.0% (2.0–3.9%)	9.7% (8.0–11.3%)	0.2% (0.0–0.4%)
Past use	24.0% (21.7–26.3%)	6.5% (5.1–7.9%)	0.3% (0.0–0.6%)
Daily use	19.5% (17.4–21.7%)	5.0% (3.8–6.2%)	0.3% (0.0–0.6%)
Past experimentation	24.6% (22.3–26.9%)	7.2% (5.7–8.7%)	6.0% (4.8–7.2%)
Total	54.1% (51.4–56.8%)	24.1% (21.7–26.5%)	6.5% (5.3–7.7%)
Nicotine-containing e-cigarette use			
Current use	5.0% (3.8–6.2%)	9.4% (7.7–11.1%)	0.0% (0.0–0.0%)
Past use	20.9% (18.7–23.1%)	4.7% (3.5–5.9%)	0.1% (0.0–0.2%)
Past experimentation	18.9% (16.8–21.0%)	4.4% (3.2–5.6%)	2.7% (1.9–3.5)
Total	44.8% (42.1–47.5%)	18.6% (16.4–20.8%)	2.9% (2.1–3.7%)

Among ever e-cigarette users who were current smokers, past experimentation and past e-cigarette use were more prevalent compared to current e-cigarette use (*χ*^2^ = 197.2, *P* < 0.001). Among ever e-cigarette users who were former smokers, current e-cigarette use was more prevalent than former e-cigarette use and past e-cigarette experimentation (*χ*^2^ = 15.1, *P* = 0.001). Among ever e-cigarette users who were never smokers, past e-cigarette experimentation was by far the most prevalent pattern of use (*χ*^2^ = 178.6, *P* < 0.001). Characteristically, 92.9% of never smoking ever e-cigarette users reported past experimentation only. The majority of current and former smokers reported daily current and past e-cigarette use. For never smokers, all current and past e-cigarette users reported daily use but the overall prevalence of e-cigarette use was very low in this population subgroup.

Similar patterns of use were observed for nicotine-containing e-cigarettes, with approximately 90% of current e-cigarette users who reported being current or former smokers were using nicotine. However, such use was very rare among never smokers. Only 1 never-smoking participant reported being current, and 2 reported being former nicotine containing e-cigarette users.

### Perception of e-cigarette harmfulness

The perception of e-cigarette harmfulness for the whole population and according to the smoking status is presented in Table 3. The most prevalent perceptions were that e-cigarettes are equally or less harmful than tobacco cigarettes. A remarkably low proportion perceived e-cigarettes as a lot less harmful than cigarettes, including among current smokers, and a substantial proportion responded that they did not know. While no statistically significant differences were observed in the perceptions that e-cigarettes are a lot more or more harmful than smoking, a larger proportion of never smokers considered e-cigarettes less harmful than smoking. Additionally, more former smokers considered e-cigarettes a lot less harmful than smoking or absolutely harmless and more current smokers were uncertain about the harmfulness of e-cigarettes.

**Table 3:** Perception of e-cigarette harmfulness

	Current smokers	Former smokers	Never smokers	Total	*P* ^1^
	% (95%CI) (weighted)				
Perception					
A lot more harmful than smoking	3.1% (2.2–4.0%)	2.6% (1.7–3.5%)	1.9% (1.2–2.6%)	2.5% (2.0–3.0%)	0.180
More harmful than smoking	7.8% (6.3–9.2%)	6.1% (4.7–7.5%)	5.4% (4.3–6.5%)	6.4% (5.6–7.2%)	0.074
Equally harmful to smoking	28.6% (26.2–31.0%)	21.2% (18.9–23.5%)	26.3% (24.1–28.5%)	25.5% (24.2–26.8%)	0.005
Less harmful than smoking	30.9% (28.4–33.4%)	30.0% (27.4–32.6%)	36.3% (33.9–38.7%)	32.7% (31.3–34.1%)	< 0.001
A lot less harmful than smoking	4.6% (3.5–5.7%)	6.8% (5.4–8.2%)	5.0% (3.9–6.1%)	5.4% (4.7–6.1%)	0.008
Absolutely harmless	0.7% (0.3–1.1%)	2.7% (1.8–3.6%)	1.0% (0.5–1.5%)	1.4% (1.0–1.8%)	< 0.001
Don’t know	20.5% (18.3–22.7%)	18.8% (16.6–21.0%)	14.7% (12.9–16.5%)	17.8% (16.6–19.0%)	0.001

^1^Comparison between current, former and never smokers (*χ*^2^ test)

### Correlates of current and ever e-cigarette use

The results of the logistic regression analyses are presented in Table 4. Being current or former smoker were the strongest correlates of ever e-cigarette use. Age younger than 55, male gender and perception of low harm for e-cigarettes were also correlates of ever e-cigarette use. University education and being married/living with partner were negatively associated with ever e-cigarette use.

**Table 4:** Correlates of ever and current e-cigarette use

	Ever e-cigarette use		Current e-cigarette use	
Correlates	OR (95%CI)	*P*	OR (95%CI)	*P*
Smoking status				
Never smokers (referent)				
Current smokers	33.38 (25.20–44.21)	< 0.001	30.82 (10.21–93.01)	< 0.001
Ex-smokers	9.05 (6.81–12.04)	< 0.001	69.33 (23.12–207.90)	< 0.001
Age (years)				
55 and older (referent)				
18–24	5.74 (3.82–8.63)	< 0.001	1.83 (0.89–3.80)	0.103
25–39	3.50 (2.68–4.59)	< 0.001	2.50 (1.57–4.0)	< 0.001
40–54	1.68 (1.33–2.13)	< 0.001	1.19 (0.77–1.84)	0.444
Gender				
Female (referent)				
Male	1.22 (1.02–1.45)	0.029	1.46 (1.06–2.01)	0.020
Education				
High school or less (referent)				
Technical education	0.93 (0.69–1.27)	0.664	0.92 (0.56–1.53)	0.754
University education	0.71 (0.58–0.88)	0.001	0.53 (0.37–0.77)	0.001
Postgraduate education	0.82 (0.60–1.13)	0.226	0.61 (0.35–1.06)	0.079
Marital status				
Single (referent)				
Married/living with partner	0.70 (0.56–0.89)	0.003	0.98 (0.65–1.47)	0.910
Divorced, widowed or other	0.86 (0.60–1.25)	0.432	1.54 (0.80–2.95)	0.196
Financial situation				
Very bad/bad (referent)				
Not good	0.99 (0.82–1.21)	0.950	0.75 (0.53–1.06)	0.101
Good	1.30 (0.97–1.74)	0.075	1.02 (0.62–1.67)	0.947
Perceived of e-cigarette harmfulness				
A lot more harmful/more harmful/equally harmful/DK (referent)				
Less harmful/a lot less harmful/harmless	2.81 (2.34–3.37)	< 0.001	6.28 (4.35–9.07)	< 0.001

For current e-cigarette use, being current or former smoker were also by far the strongest correlates. Age 25–39 years, male gender and perception of low harm for e-cigarettes were also correlates of current e-cigarette use. University education was negatively associated with current e-cigarette use.

## Discussion

This study presents the first evidence on prevalence and patterns of e-cigarette use in Greece, a European country with a very high prevalence of smoking. The study identified almost universal awareness of e-cigarettes, while more than 1 out of 4 participants reported ever e-cigarette use. However, current e-cigarette use was substantially lower and mainly confined to current and former smokers, with the latter representing the majority of current e-cigarette users.

The prevalence of current e-cigarette use in Greece is similar to the UK, where it was recently reported that 5.8% of the population is using e-cigarettes [25]. In the EU in 2014, current e-cigarette use was reported by 1.8% of the population, with 60% of current users reporting daily use [24, 26]. In the United States (US) in 2014, the prevalence of current e-cigarette use (defined as any past 30-day use) was 5.5%, with only 21.3% of those reporting daily use. These data identify higher prevalence and more intense use by the Greek population compared to the average use in the EU and the US while use prevalence is similar compared to the UK, a country with a positive environment for e-cigarettes among the public health community. There has been a lot of debate about the definition of current e-cigarette use in the US, defined as any past 30-day use, because it includes a lot of recent experimenters [27]. Additionally, frequency of e-cigarette use is positively associated with both quit attempts and quit success [28]. Considering that frequency of use seems to vary considerably between different populations, it is important to record such information in population surveys in order to make proper comparisons. Frequency of use and nicotine use are among the principle items that are recommended as measures included in population surveys examining e-cigarette use [29].

An important finding of this study is the high proportion of current e-cigarette users who were former smokers. Being current and former smoker were the strongest correlates of e-cigarette use, with a particularly strong association observed for former smokers. E-cigarettes were also by far the most popular smoking cessation aid among current daily smokers. Similar findings have been observed in the UK where the proportion of e-cigarette users being former smokers surpassed current smokers for the first time in 2017 [25] while, over the past 3 years, e-cigarettes have replaced nicotine replacement therapies as the most popular alternative to smoking for people trying to quit [30]. Current e-cigarette use was remarkably uncommon among Greek never smokers. These findings provide indirect evidence that e-cigarette use is maintained because it helps smokers quit, while e-cigarettes are not attractive to never smokers. This represents a desirable effect from a public health perspective and appears reassuring for the potential threat that e-cigarettes act as a gateway to smoking. However, there are inherent limitations in cross-sectional surveys when addressing such research questions, which are discussed below.

An important issue raised by the study herein is the overestimation of e-cigarette harmfulness relative to smoking. Only about 1 in 20 participants correctly considered e-cigarettes as a lot less harmful than smoking while perceptions of low harmfulness was a correlate of ever and current e-cigarette use. Such misconceptions were more pronounced among smokers. Several studies have shown a growing misconception in the general population about the relative risks of e-cigarettes [25, 31–33]. Brose et al. found that perception of low harm predicted subsequent use of e-cigarettes in a cohort of current and former smokers [31]. A cross-sectional survey of dedicated vapers found that perception of low harm was independently associated with being exclusive e-cigarette user rather than dual user [34]. Since overestimating their harmfulness may discourage smokers from using e-cigarettes in an effort to quit smoking, there is a need to provide accurately and reliable information about the risks of smoking and e-cigarette use, particularly to smokers [19, 35]. Studies need to examine the factors associated with such misconceptions. For example, media articles that largely exaggerate the potential adverse health effects of e-cigarettes and provide no comparison with the effects of smoking, a phenomenon that is frequently observed in Greece, could be responsible for providing misleading messages to smokers [19].

Limitations applicable to this study are the inherent weaknesses of cross-sectional studies. Associations cannot be interpreted as causation, while lack of temporality does not allow for determining if e-cigarette use preceded smoking cessation in former smokers. The smoking status was self-reported and not objectively verified, which is common for large population studies. Still, the study is valuable in showing for the first time that e-cigarette use in the Greek population is adopted by smokers only and is minimal among never smokers, largely rejecting the concerns about gateway to smoking effects.

## Conclusions

In conclusion, current e-cigarette use in Greece is predominantly confined among current and former smokers. In never smokers, minimal experimentation and almost no current use was observed. The majority of current e-cigarette users reported being former smokers, while e-cigarettes are the most popular smoking cessation aid among current daily smokers over the past 3 years. The findings suggest a positive public health impact of e-cigarettes in Greece, which is particularly important for a country having a high prevalence of smoking, and provide reassurance that e-cigarettes are not popular among never smokers. However, major misconceptions about the relative risks of e-cigarettes compared to smoking exist in the population, and this needs to be addressed.

## Additional file

Additional file 1: **Table S1.** Demographics of study participants. (DOCX 14 kb)

## Abbreviations

EU: European Union
US: United States
