# Supplementary material for: Electronic cigarette use in Greece: an analysis of a representative population sample in Attica prefecture
Source: Harm Reduct J. 2018 Apr 13;15:20. doi: 10.1186/s12954-018-0229-7 (PMC5899338; doi:10.1186/s12954-018-0229-7)
Supplement: Supplementary file 1 — Table S1. Demographics of study participants. (DOCX 14 kb) [file 12954_2018_229_MOESM1_ESM.docx]

**Table S1.** Demographics of study participants.

| **Characteristic** | **Proportion (95%CI)** |
| --- | --- |
| Gender |  |
| Males | 47.9% (46.4-49.5%) |
| Females | 52.1 % (50.5-53.6%) |
| Age |  |
| 18-24 | 9.7% (8.8-10.6%) |
| 25-39 | 28.3% (26.9-29.7%) |
| 40-55 | 28.3% (26.9-29.7%) |
| > 55 | 33.7% (32.2-35.2%) |
| Education |  |
| High school or less | 38.4% (36.9-39.9%) |
| Technical education | 8.9% (8.0-9.8%) |
| University education | 42.4% (40.9-43.9%) |
| Postgraduate education | 10.2% (9.3-11.1%) |
| Residence |  |
| Athens | 70.4% (69.0-71.8%) |
| Piraeus | 11.9% (10.6-12.6%) |
| Eastern Attica | 11.6% (10.6-12.6%) |
| Western Attica | 4.8% (4.1-5.5%) |
| Marital status |  |
| Single | 33.0% (31.6-34.4%) |
| Married/living with partner | 55.9% (54.4-57.4%) |
| Divorced/widowed | 10.4% (9.5-11.3%) |
| Financial self-assessment |  |
| Very bad/bad | 37.9% (36.4-39.4%) |
| Not good | 48.7% (47.2-50.2) |
| Good | 11.8% (10.8-12.8%) |
